# Genitourinary symptoms and sexual dysfunction in women with premature ovarian insufficiency: a cross-sectional study with age-comparable controls

**DOI:** 10.3389/fendo.2026.1880566

**Published:** 2026-06-22

**Authors:** Zijie Fu, Xueping Liu, Wei Guo, Yahui Bian, Tongyao Geng, Bianling Xu, Jiabei Hao, Xiaodong Li

**Affiliations:** Department of Gynecology, The First Hospital of Hebei Medical University, Shijiazhuang, China

**Keywords:** female sexual function index, genitourinary syndrome, premature ovarian insufficiency, sexual function, vulvovaginal dryness

## Abstract

**Purpose:**

This study aimed to characterize genitourinary symptoms and sexual function in women with premature ovarian insufficiency (POI) and to compare these findings with those in age-comparable women with regular menstruation.

**Methods:**

This questionnaire-based cross-sectional study identified women with POI who attended the First Hospital of Hebei Medical University between April 2022 and October 2025. Eligible women were invited to complete questionnaires between October and December 2025 through telephone interviews or outpatient follow-up visits. General genitourinary symptoms were assessed using a structured questionnaire. Female sexual function was evaluated using the Female Sexual Function Index (FSFI), and urinary incontinence-related outcomes were assessed using the International Consultation on Incontinence Questionnaire (ICIQ). Multivariable linear regression analysis was performed to identify factors associated with FSFI total score, with sensitivity analysis excluding the FSFI lubrication domain.

**Results:**

Among 207 invited women with POI, 149 returned valid questionnaires, yielding a valid response rate of 72.0%. In addition, 51 age-comparable women with regular menstruation completed questionnaires as controls. Compared with controls, women with POI had more frequent vulvovaginal dryness, whereas vulvovaginal itching, vulvovaginal burning, and individual lower urinary tract symptoms did not differ significantly. Neither the ICIQ total score nor the proportion of moderate-to-severe urinary incontinence differed significantly between groups. Among women with a history of sexual activity, those with POI had lower FSFI total scores and a higher prevalence of female sexual dysfunction, with impairment in arousal, lubrication, orgasm, satisfaction, and pain. In multivariable linear regression analysis, vulvovaginal dryness was independently associated with lower FSFI total score (B = -0.804, 95% CI: -1.152 to -0.457, *P* < 0.001). This association remained significant after excluding the lubrication domain in sensitivity analysis (B = -0.624, 95% CI: -0.910 to -0.338, *P* < 0.001).

**Conclusion:**

Women with POI showed a clinically meaningful early genitourinary symptom burden characterized predominantly by vulvovaginal dryness and multidimensional sexual dysfunction, whereas overt urinary incontinence severity was not significantly increased. Vulvovaginal dryness showed the most consistent association with impaired sexual function and may serve as a clinical clue to sexual dysfunction in women with POI.

## 1 Introduction

Premature ovarian insufficiency (POI) is defined as loss of ovarian follicular activity before the age of 40 years and is commonly characterized by menstrual disturbance and infertility. POI is associated not only with symptoms related to estrogen deficiency, but also with osteoporosis, increased cardiovascular disease risk, cognitive decline, mental health problems, and impaired sexual health, all of which may substantially reduce quality of life (1–3). Accordingly, the evaluation of women with POI should extend beyond endocrine and reproductive outcomes to include broader domains of long-term health and well-being.

Genitourinary syndrome of menopause (GSM) refers to a collection of vulvovaginal, sexual, and lower urinary tract symptoms related to reduced sex steroid exposure, particularly estrogen deficiency. Typical manifestations include vulvovaginal dryness, itching, burning, dyspareunia, reduced lubrication, urinary urgency, urinary frequency, dysuria, recurrent urinary tract infection, and urinary incontinence, all of which may adversely affect daily functioning, sexual health, and quality of life (4–6). However, current definitions, epidemiological evidence, and management recommendations for GSM have largely been derived from naturally postmenopausal populations, in whom the condition is common but remains underdiagnosed and undertreated (7, 8).

Because POI represents a state of early hypoestrogenism, women with POI may also develop GSM-like symptoms at a much younger age (9, 10). However, due to differences in chronological age, reproductive history, pelvic floor background, and duration of estrogen deprivation, the clinical pattern of genitourinary complaints in POI may not be entirely consistent with that observed in naturally postmenopausal women. Previous studies in POI have focused predominantly on diagnosis, hormone therapy, and long-term sequelae. Even when sexual health has been addressed, the emphasis has usually been on overall sexual function rather than on the broader spectrum of genitourinary symptoms. Recent evidence indicates that women with POI have poorer sexual function and a higher risk of impaired sexual function than controls; however, studies integrating general genitourinary symptoms and sexual function within an age-comparable comparative framework remain limited (11–15).

The present study aimed to systematically characterize genitourinary symptoms and sexual function in women with POI, compare these findings with those in age-comparable women with regular menstruation, and further explore the associations between genitourinary symptoms and sexual function.

## 2 Materials and methods

This questionnaire-based cross-sectional study identified women who attended the First Hospital of Hebei Medical University between April 2022 and October 2025 and had been diagnosed with POI before the age of 40 years. The study was approved by the Ethics Committee of the First Hospital of Hebei Medical University on December 8, 2023 (Approval No.2023-00049). All participants provided written informed consent prior to enrollment.

### 2.1 Participants

Eligible women were subsequently contacted and invited to complete the study questionnaires between October and December 2025 through telephone interviews or outpatient follow-up visits. POI diagnosis was confirmed through review of medical records according to the ESHRE guideline as oligo/amenorrhea for at least 4 months before the age of 40 years, accompanied by elevated basal serum follicle-stimulating hormone (FSH) levels >25 IU/L on two separate occasions at least 4 weeks apart (16). Therefore, some participants could be older than 40 years at the time of questionnaire assessment.

Exclusion criteria included: severe diseases of other systems; cognitive impairment; substance abuse or dependence within the previous 3 months; acute inflammatory conditions; pregnancy or lactation; and participation in another study that could conflict with the present study. Age-comparable women with regular menstruation and no known history of POI were recruited as controls from women attending the gynecology outpatient clinic during the same survey period.

### 2.2 Data collection and questionnaires

Demographic and reproductive variables collected included age, body mass index (BMI), age at menarche, gravidity, parity, sexual activity, time since POI diagnosis and current hormone replacement therapy (HRT) use. Time since POI diagnosis was calculated as the interval between the confirmed diagnosis of POI and questionnaire completion. Current HRT use was defined as the use of systemic hormone therapy at the time of questionnaire assessment.

A structured questionnaire was used to assess genitourinary symptoms, including vulvovaginal dryness, itching, burning, dyspareunia, and lower urinary tract symptoms including urinary frequency, urinary urgency, dysuria, and recurrent lower urinary tract infection. The severity of vulvovaginal dryness, itching, and burning was quantified using a 10-point visual analog scale (VAS), with higher scores indicating greater symptom severity or bother. In the present study, the VAS was used as a symptom-severity measure rather than as a pain-only scale (17).

Sexual function was assessed using the Female Sexual Function Index (FSFI), a 19-item questionnaire comprising six domains: desire, arousal, lubrication, orgasm, satisfaction, and pain (18). FSFI-based analyses were restricted to women who reported a history of sexual activity and completed the FSFI questionnaire. Female sexual dysfunction (FSD) was defined using the established FSFI cut-off (19). Urinary symptoms were assessed using the International Consultation on Incontinence Questionnaire (ICIQ), and both the total score and the proportion of moderate-to-severe urinary incontinence were analyzed (20).

### 2.3 Survey procedure

To reduce bias, the investigators received standardized training before data collection, and all questionnaires were administered using uniform instructions. The survey included the general information form, the genitourinary symptom questionnaire, the FSFI, and the ICIQ. Before the survey, the purpose and significance of the study were explained to each participant, and informed consent was obtained.

### 2.4 Sample size

The sample size was determined by the number of eligible women with POI who could be contacted and completed valid questionnaires during the study period. Age-comparable controls were recruited as a reference group during the same period; therefore, the group sizes reflected the actual recruitment process of this observational study rather than a prespecified allocation ratio.

### 2.5 Statistical analyses

Statistical analyses were performed using SPSS 27.0 software. Continuous variables were tested for normality and are presented as mean ± standard deviation or median (interquartile range), as appropriate, whereas categorical variables are presented as numbers and percentages. Group comparisons were performed using the independent-samples *t* test, Mann–Whitney U test, chi-square test, or Fisher’s exact test, as appropriate. Multivariable linear regression analysis was performed to identify factors associated with FSFI total score. Variables included in the multivariable model were selected based on clinical relevance and prior evidence. *P* < 0.05 was considered statistically significant.

## 3 Results

Among 207 eligible women with POI who were invited to participate, 149 completed valid questionnaires, yielding a valid response rate of 72.0%. The remaining 58 women did not provide valid questionnaire data, including 1 with incomplete survey information, 53 who could not be reached, and 4 who declined participation. Comparison of available baseline characteristics showed no significant differences between POI responders and non-responders in age, BMI, menstrual status, gravidity, parity, or etiology of POI, suggesting a relatively low risk of major non-response bias. In addition, 51 eligible age-comparable controls with regular menstruation and no known history of POI were invited from the gynecology outpatient clinic during the same survey period, and all completed the questionnaire.

The two groups were broadly comparable in age and BMI, although some reproductive characteristics differed between groups. Compared with controls, women with POI were less likely to report spontaneous menarche and had a later age at menarche, whereas gravidity, parity, and sexual activity did not differ significantly between groups. Among women with POI, the median time since POI diagnosis was 6.0 years. Current HRT use was reported by 119 women with POI (79.9%), most commonly as oral estradiol valerate 2 mg or oral estradiol 2 mg/dydrogesterone 10 mg, whereas none of the controls reported HRT use; however, detailed information on HRT duration, dose adjustment, and treatment adherence was not consistently available. With respect to genitourinary symptoms, vulvovaginal dryness was more common in the POI group, while differences in vulvovaginal itching, vulvovaginal burning, and individual lower urinary tract symptoms did not reach statistical significance. Neither the ICIQ total score nor the proportion of moderate-to-severe urinary incontinence differed significantly between groups, suggesting that urinary incontinence may not represent the predominant genitourinary clinical burden in young women with POI (**Table 1**).

**Table 1:** Clinical characteristics, genitourinary symptoms, and ICIQ-based outcomes in women with POI and controls.

Variable	POI (n=149)	Controls (n=51)	*P*
Demographic and clinical characteristics			
Age, years	30.0 (15.0)	29.0 (13.0)	0.894
BMI, kg/m²	22.15 (4.48)	21.48 (4.72)	0.790
Spontaneous menarche, n (%)	101 (67.8%)	51 (100%)	<0.001
Age at menarche	14.0 (3.0)	13.0 (2.0)	0.002
Gravidity	0 (2)	0 (2)	0.679
Parity	0 (1)	0 (1)	0.461
Sexual activity, n (%)	88 (59.1%)	36 (70.6%)	0.143
Time since POI diagnosis, years	6.0 (4.0)	NA	NA
Current HRT use, n (%)	119 (79.9%)	0 (0.0%)	<0.001
Vulvovaginal symptoms			
Vulvovaginal dryness, n (%)	60 (40.3%)	11 (21.6%)	0.016
Vulvovaginal itching, n (%)	52 (34.9%)	22 (43.1%)	0.293
Vulvovaginal burning, n (%)	20 (13.4%)	3 (5.9%)	0.145
Urinary symptoms			
Urinary frequency ≥8/day, n (%)	4 (2.7%)	1 (2.0%)	1.000
Urinary urgency, n (%)	8 (5.4%)	3 (5.9%)	1.000
Dysuria, n (%)	2 (1.3%)	1 (2.0%)	1.000
Recurrent lower urinary tract infection, n (%)	10 (6.7%)	3 (5.9%)	1.000
ICIQ total score	0.00 (0)	0.00 (3)	0.086
Moderate to severe urinary incontinence, n (%)	5 (3.4%)	3 (5.9%)	0.333

Data are presented as median (interquartile range) or n (%), as appropriate. ICIQ, International Consultation on Incontinence Questionnaire; POI, premature ovarian insufficiency; BMI, body mass index; HRT, hormone replacement therapy; NA, not applicable.

Among women who reported a history of sexual activity, the FSFI total score was significantly lower in the POI group and the prevalence of FSD was higher. Domain-specific analyses showed significant impairment in arousal, lubrication, orgasm, satisfaction, and pain, whereas the desire domain did not differ significantly between groups. These findings indicate that sexual impairment in women with POI is multidimensional and is particularly characterized by reduced lubrication, impaired sexual response, and increased intercourse-related discomfort (**Table 2**).

**Table 2:** Comparison of FSFI domain scores, total score, and prevalence of FSD between women with POI and controls.

Variable	POI (n=88)	Controls (n=36)	*P*
Desire	3.6 (1.2)	3.6 (1.7)	0.111
Arousal	3.6 (1.5)	3.9 (1.2)	<0.001
Lubrication	4.2 (1.2)	5.4 (1.4)	<0.001
Orgasm	3.6 (1.2)	4.4 (1.5)	<0.001
Satisfaction	3.6 (1.2)	4.8 (1.6)	<0.001
Pain	4.4 (1.6)	5.2 (1.2)	<0.001
FSFI total score	18.30 (23.8)	25.5 (29.2)	<0.001
FSD prevalence, n (%)	65 (73.9%)	14 (38.9%)	<0.001

Data are presented as median (interquartile range) or n (%). FSD was defined as FSFI total score <26.55. FSFI, Female Sexual Function Index; FSD, female sexual dysfunction; POI, premature ovarian insufficiency.

In multivariable linear regression analysis among women with POI, vulvovaginal dryness remained independently associated with lower FSFI total score after adjustment for age, BMI, parity, current HRT use, and other vulvovaginal symptoms (95% CI: -1.152 to -0.457, *P* < 0.001). Age was also independently associated with lower FSFI total score (95% CI: -0.412 to -0.059, *P* = 0.010). By contrast, BMI, vulvovaginal itching, vulvovaginal burning, and current HRT use were not significantly associated with FSFI total score in the adjusted model (**Table 3**).

**Table 3:** Multivariable linear regression analysis of factors associated with overall sexual function among women with POI.

Variable	B coefficient	95% CI	*P*
Age	-0.236	(-0.412, -0.059)	0.010
BMI	0.237	(-0.054, 0.528)	0.110
Vulvovaginal dryness (VAS, per 1-point increase)	-0.804	(-1.152, -0.457)	<0.001
Vulvovaginal itching (VAS, per 1-point increase)	-0.398	(-0.993, 0.197)	0.187
Vulvovaginal burning (VAS, per 1-point increase)	0.108	(-0.726, 0.942)	0.798
Parity	1.144	(-0.013, 2.300)	0.052
Current HRT use (yes *vs* no)	0.168	(-1.722, 2.059)	0.860

BMI, body mass index; CI, confidence interval; VAS, visual analog scale; HRT, hormone replacement therapy.

Considering the potential conceptual overlap between vulvovaginal dryness and the lubrication domain of the FSFI, a sensitivity analysis was performed using a modified FSFI score excluding the lubrication domain as the dependent variable. Vulvovaginal dryness remained independently associated with lower modified FSFI scores (95% CI: -0.910 to -0.338, *P* < 0.001), and age also remained statistically significant (95% CI: -0.343 to -0.052, *P* = 0.008) (**Supplementary Table 1**).

## 4 Discussion

In this cross-sectional study, women with POI exhibited a clinically meaningful pattern of early genitourinary symptom burden when compared with age-comparable women with regular menstruation. Importantly, not every genitourinary complaint was uniformly increased in POI; rather, the symptom burden in this young hypoestrogenic population was concentrated in the vulvovaginal and sexual domains. Women with POI more frequently reported vulvovaginal dryness, had poorer overall sexual function, and showed a substantially higher prevalence of female sexual dysfunction, whereas lower urinary tract symptoms and the severity of overt urinary incontinence did not differ significantly from those of controls. These findings suggest that, in young women with POI, the clinical consequences of estrogen deprivation on the genitourinary system may be expressed more prominently as vulvovaginal discomfort and impaired sexual function than as urinary symptoms (1–4).

Current epidemiological descriptions and management recommendations for GSM are largely derived from naturally postmenopausal populations. By contrast, POI represents an early hypoestrogenic state in younger women, whose reproductive characteristics, pelvic floor baseline, and psychosocial context differ substantially from those of postmenopausal women. Therefore, the genitourinary manifestations of estrogen deprivation in POI should not be assumed to fully correspond to the postmenopausal GSM phenotype. Although our study did not directly compare women with POI with naturally postmenopausal women, the finding that vulvovaginal and sexual symptoms were more prominent than urinary symptoms suggests that the clinical expression of hypoestrogenism in POI may have a distinct pattern. These results extend the GSM framework to a younger hypoestrogenic population and highlight the need to consider life stage and background pelvic floor risk when interpreting genitourinary symptoms in women with POI (21–23).

Those with POI had a lower FSFI total score and a higher prevalence of female sexual dysfunction. Importantly, the impairment was not confined to a single domain: arousal, lubrication, orgasm, satisfaction, and pain were all adversely affected. These findings suggest that sexual dysfunction in POI is a multidimensional disturbance involving both sexual response and intercourse-related comfort. This interpretation is in line with previous observational studies and with the recent systematic review and meta-analysis showing poorer overall sexual function in women with POI, particularly in the domains of arousal, lubrication, satisfaction, and pain (9–15).

We further evaluated which vulvovaginal symptoms were independently associated with sexual function. After adjustment for age, BMI, parity, current HRT use, and other vulvovaginal symptoms, vulvovaginal dryness remained independently associated with lower FSFI total score. This association is clinically plausible, as vulvovaginal dryness may not only reflect reduced lubrication but may also increase intercourse-related friction and discomfort, contribute to pain, reduce sexual confidence, and be associated with lower arousal and satisfaction. To address the potential conceptual overlap between vulvovaginal dryness and the lubrication domain of the FSFI, we conducted a sensitivity analysis excluding the lubrication domain, and the association remained significant. This sensitivity analysis supports the robustness of the observed association and suggests that the clinical relevance of vulvovaginal dryness may extend beyond the lubrication-related dimension, although causality cannot be inferred from the present cross-sectional data. From a clinical perspective, vulvovaginal dryness may serve as an identifiable symptom marker of poorer sexual function in women with POI, highlighting the need for comprehensive sexual health assessment and timely symptom management.

Unlike vulvovaginal symptoms and sexual function outcomes, urinary symptoms and ICIQ-based outcomes did not show a significant POI-related increase compared with age-comparable controls. No significant between-group differences were observed in individual lower urinary tract symptoms, ICIQ total score, or the proportion of moderate-to-severe urinary incontinence. Urinary symptoms and pelvic floor dysfunction are multifactorial and dependent on life-stage-related pelvic floor background (14, 24, 25). Evidence from urogynecologic research indicates that urinary incontinence and pelvic floor dysfunction are influenced by hormonal milieu as well as mechanical load, parity, mode of delivery, obesity, aging, and pelvic floor muscle and connective tissue changes (26). In the present study, the median time since POI diagnosis was 6.0 years (Q1–Q3, 4.0–8.0; range, 0–23 years). Although most women with POI reported current HRT use, detailed information on HRT duration and adherence was not consistently available; therefore, cumulative hypoestrogenic exposure could not be accurately determined. Accordingly, the lack of significant between-group differences in urinary symptoms should be interpreted cautiously, as we could not determine whether a longer duration of untreated estrogen deficiency would be associated with a greater urinary symptom burden. In addition, both groups were relatively young and had low parity, suggesting a limited cumulative burden of childbirth-related pelvic floor injury and aging-related lower urinary tract changes in both groups. This may also partly explain why overt urinary symptoms and ICIQ-based urinary incontinence severity were not significantly increased in the POI group despite the presence of hypoestrogenism. Further longitudinal studies with detailed assessment of estrogen deprivation and treatment exposure are needed to clarify the long-term impact of POI on urinary symptoms. In addition, the baseline differences in spontaneous menarche and age at menarche between the POI and control groups should be considered when interpreting our findings. These variables may reflect differences in pubertal development, early-life endocrine milieu, and underlying ovarian function, and may be closely related to the development of POI itself. Population-based evidence indicates that pelvic floor disorders are strongly influenced by life-course and reproductive factors, including age, parity, and BMI (27). Emerging evidence also suggests that age at menarche may be associated with lower urinary tract symptoms. Al-Mehaisen et al. reported that later menarche was associated with a higher prevalence of urgency and urgency urinary incontinence, whereas earlier menarche was associated with an increased likelihood of nocturia in young women (28). Huang et al. found that earlier age at menarche was associated with an increased risk of overactive bladder, with BMI potentially mediating part of this association (29). Taken together, these findings suggest that reproductive developmental factors may influence subsequent urogenital health. However, because menarche-related characteristics may partly represent the developmental background of POI rather than independent confounders, we did not include them as additional adjustment variables to avoid potential overadjustment. Nevertheless, residual confounding related to developmental and reproductive characteristics cannot be entirely excluded.

This study has several strengths. First, the inclusion of age-comparable women with regular menstruation as controls enabled us to assess the genitourinary symptom burden associated with POI without conflating it with the older age profile of natural menopause. Second, by evaluating general genitourinary symptoms, FSFI outcomes, and urinary incontinence-related outcomes within the same study framework, we were able to provide a relatively comprehensive description of the genitourinary symptom profile in women with POI. Third, we further explored the association between vulvovaginal symptoms and sexual function and found that vulvovaginal dryness was associated with poorer overall sexual function. Given that vulvovaginal dryness is readily identifiable in clinical practice, it may represent an important clinical clue to sexual dysfunction and support earlier assessment and timely intervention in women with POI.

Several limitations of this study should be acknowledged. First, because of the cross-sectional design, causal relationships cannot be inferred. Second, ovarian function in the control group was not biochemically confirmed. Although controls had regular menstruation and no known history of POI, subclinical ovarian dysfunction could not be entirely excluded. If present, this could have slightly skewed the comparative findings by increasing genitourinary symptom burden or reducing sexual function in the control group, thereby potentially attenuating the observed differences between groups. Third, although current HRT use, regimen, dose and time since POI diagnosis were recorded, detailed information on HRT duration, dose adjustment and adherence was not consistently available. Therefore, cumulative treatment exposure and cumulative hypoestrogenic exposure could not be accurately assessed, limiting interpretation of the relationships among HRT use, genitourinary symptoms, and sexual function outcomes. Accordingly, the lack of a significant association between current HRT use and FSFI scores should be interpreted cautiously. Finally, FSFI-based analyses were restricted to women with a history of sexual activity who completed the FSFI questionnaire. Although this restriction was methodologically necessary, it may limit the generalizability of the sexual function findings to all women with POI, particularly those without sexual experience.

In the future, artificial intelligence-assisted approaches may be explored to integrate patient-reported genitourinary symptoms, sexual function measures, endocrine profiles, treatment exposure, and other clinical characteristics to identify women with POI who are at higher risk of genitourinary and sexual health problems. Such approaches may support earlier recognition, individualized follow-up, and more personalized symptom management. However, their clinical application will require standardized data collection, external validation, and careful implementation in real-world practice (30).

## 5 Conclusion

In conclusion, women with POI exhibited a clinically meaningful early burden of genitourinary symptoms, most prominently characterized by vulvovaginal dryness and multidimensional sexual dysfunction. Among the vulvovaginal symptoms evaluated, vulvovaginal dryness showed the most consistent association with impaired sexual function. These findings suggest that, in the clinical management of POI, vulvovaginal dryness may be regarded as an important clinical clue for identifying women who may have sexual dysfunction and therefore warrant further sexual health assessment and timely intervention.

## Data Availability

The raw data supporting the conclusions of this article will be made available by the authors, without undue reservation.
