# Supplementary material for: Genitourinary symptoms and sexual dysfunction in women with premature ovarian insufficiency: a cross-sectional study with age-comparable controls
Source: Front Endocrinol (Lausanne). 2026 Jun 22;17:1880566. doi: 10.3389/fendo.2026.1880566 (PMC13333530; doi:10.3389/fendo.2026.1880566)
Supplement: Supplementary file 1 [file Table1.docx]

**Table S1** Multivariable linear regression of factors associated with modified FSFI score excluding the lubrication domain among women with POI

| **Variable** | **B coefficient** | **95% CI** | ***P*** |
| --- | --- | --- | --- |
| Age | -0.197 | （-0.343，-0.052） | 0.008 |
| BMI | 0.185 | （-0.055，0.424） | 0.129 |
| Vulvovaginal dryness (VAS, per 1-point increase) | -0.624 | （-0.910，-0.338） | ＜0.001 |
| Vulvovaginal itching (VAS, per 1-point increase) | -0.355 | （-0.844，0.135） | 0.153 |
| Vulvovaginal burning (VAS, per 1-point increase) | 0.077 | （-0.610，0.763） | 0.825 |
| Parity | 0.928 | （-0.024，1.880） | 0.056 |
| Current HRT use (yes vs no) | 0.065 | （-1.491，1.622） | 0.934 |

Note: BMI, body mass index; CI, confidence interval; VAS, visual analog scale; HRT, hormone replacement therapy.
